# Protocol to distinguish pre-mRNA from mRNA in RNA-protein interaction studies

**DOI:** 10.1016/j.xpro.2025.103967

**Published:** 2025-07-22

**Authors:** Christina Zeiler, Annika Bestehorn, Pavel Kovarik

**Affiliations:** 1 Max Perutz Labs, Vienna BioCenter Campus (VBC), Dr.-Bohr-Gasse 9, 1030 Vienna, Austria; 2 University of Vienna, Max Perutz Labs, Department of Microbiology, Immunobiology and Genetics, Dr.-Bohr-Gasse 9, 1030 Vienna, Austria; 3 Vienna BioCenter PhD Program, a Doctoral School of the University of Vienna and Medical University of Vienna, 1030 Vienna, Austria

**Keywords:** Bioinformatics, Sequence analysis, Genomics, Sequencing, RNA-seq, Molecular Biology, Gene Expression

## Abstract

Transcriptome-wide studies on interactions between RNA-binding proteins (RBPs) and protein-coding RNAs in general preclude interpretations regarding RBP preference for binding to the more abundant mRNA over the less abundant pre-mRNA. Here, we present a protocol to determine the binding preference of the RBP tristetraprolin (TTP, Zfp36) for pre-mRNA versus mRNA. We describe steps for the identification and quantitation of intronic and exonic fragments in RNA bound to TTP. This protocol can potentially be applied to any RBP.

For complete details on the use and execution of this protocol, please refer to Bestehorn et al.^1^

## Highlights

• Steps for efficient pull-down of full-length RNAs bound to RBPs
• Reference genome processing and unambiguous intronic and exonic fragment counting
• Intron-exon ratio calculation and determination of RBP binding preference

## Abstract (editor highlights)

Publisher’s note: Undertaking any experimental protocol requires adherence to local institutional guidelines for laboratory safety and ethics.

## Graphical abstract

**Figure:**
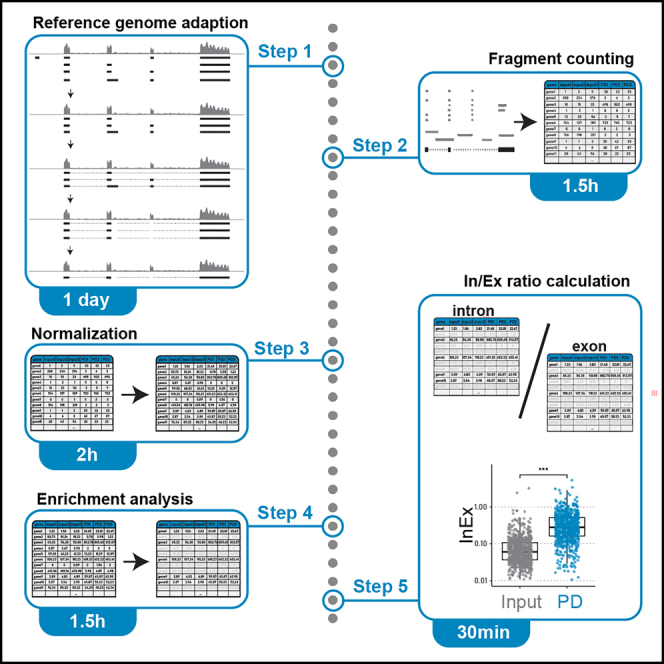

## Teaser

Transcriptome-wide studies on interactions between RNA-binding protein (RBPs) and protein-coding RNAs in general preclude interpretations regarding RBP preference for binding to the more abundant mRNA over the less abundant pre-mRNA. Here, we present a protocol to determine the binding preference of the RBP tristetraprolin (TTP, Zfp36) for pre-mRNA versus mRNA. We describe steps for the identification and quantitation of intronic and exonic fragments in RNA bound to TTP. This protocol can potentially be applied to any RBP.

## Before you begin

Total RNA-seq has previously been used to approximate transcriptional changes via pre-mRNA abundance^2^ as well as to determine posttranscriptional regulation.^3^ In contrast to these approaches, which compare total transcriptomes of two different conditions, this protocol is based on the comparison of two different fractions: RBP pull-down (PD) to Input fractions, each with a different sample complexity. The available analysis software used for differential expression analysis of samples with similar complexities is in general not suitable for the analysis of different fractions, necessitating custom solutions described in this protocol. In addition, we present a novel way for the quantitation of the relative abundance of pre-mRNA versus mRNA allowing to determine whether a given RBP prefers pre-mRNA or mRNA for binding.

In Bestehorn et al.,^1^ this protocol was applied on TTP-bound RNA in the RAW264.7 mouse macrophage cell line to consolidate the discovery that the main TTP target is pre-mRNA not mRNA. This mechanism ensures that the TTP-bound target, once spliced and exported to the cytoplasm, is degraded before it can be translated.

### Download and install the software

**Timing: 1 h**

Many steps of this protocol use previously available software. These packages can be found in the key resources table. We suggest Trim Galore, Fastp, STAR, Samtools, Deeptools and FastQC for read processing, alignment and generation of coverage tracks. In the major steps of this protocol, we use Htseq-count, Bedtools, R and tidyverse. For ease of installation, we recommend using a package manager such as conda or mamba.

Scripts for generating coverage tracks and for the major steps of this protocol are available on GitHub: https://github.com/ChristinaZeiler/InEx.

### Pull-down of RBP-bound RNA, library preparation, and sequencing

**Timing: 8 days**

This step includes the preparation of cells expressing the RBP and target RNAs, metabolic labeling of RNA with subsequent crosslinking, pull-down of RBP-RNA complexes, RNA isolation, library preparation and sequencing.

We use a RAW264.7 macrophage cell line which harbors a CRISPR/Cas9-mediated TTP deletion. These cells were engineered to inducibly express exogenous C-terminally Myc-tagged TTP from a doxycycline inducible promoter (Bestehorn et al^1^). While we have not tested the protocol for other cell lines or RBPs, we believe that it can be adapted for different cell lines and RBPs of interest (endogenous or exogeneous), provided antibodies for the pull-down step are available.

Clean workspace with an RNase decontamination solution and only use sterile filter tips throughout the experiment.

#### Day 1

1. Seed 15 million cells into 15 cm tissue culture-treated (TC) dishes in 20 mL of DMEM supplemented with 10% FBS and penicillin/streptomycin.
2. Freshly prepare Buffers A-F and sterile filter before use. Store Buffers A, B, C and F at 4°C.

***Note:*** SDS in Buffer F will precipitate at 4°C and will look turbid.

***Optional:*** Prepare Buffer G if performing optional step in step 17. Store Buffer G in aliquots at −20°C for up to 1 year.

#### Day 2

3. If using a doxycycline-inducible exogenous protein, induce expression by adding doxycycline (0.5 μg/mL for TTP in our system). Omit this step when targeting endogenous protein.

#### Day 3

4. If target RNAs need stimulation for expression, add stimulant directly into the TC dish. For the induction of TTP RNA target expression, we added 10ng/mL lipopolysaccharide (LPS) for 5 h 18 h after doxycycline treatment.
5. Meanwhile, prepare magnetic beads if targeting endogenous protein: bind antibody to magnetic protein A/G beads. Skip this step when working with Myc-tagged exogenous protein.
  a. Wash magnetic A/G beads 3 times in Buffer A.
  b. Add suitable antibody dilution (test the dilution in pilot experiments) in Buffer A to beads and rotate gently for 1–2 h at 20°C–25°C,
  c. Wash beads in Buffer A 3 times and keep on ice until use.

***Note:*** Wash beads as mastermix rather than individual samples to limit variance of bead volume between samples. In the last wash, distribute beads into microtubes. In this step protease inhibitor cocktail in Buffer A can be omitted since commercially purchased beads and antibodies are guaranteed by the manufacturer to be protease free.

6. Add 100 μM 4-thiouridine (4sU) directly into the TC dish 5h after induction of target RNA expression, to allow incorporation of 4sU into freshly transcribed RNA and continue to incubate at 37°C 5% CO2 for 1 h.

***Note:*** Turn off the lights in tissue culture hood to prevent undesired reaction of light sensitive 4sU. After labeling, place TC dishes on ice and prevent light exposure as much as possible (use ice box with lid).

7. Wash TC dishes 2 times with excess of cold PBS (>10 mL).

***Note:*** From here on, sterile working conditions are no longer necessary.

For RAW macrophages and other very adherent cells, PBS can be gently poured onto a TC dish, shortly swirled around by tilting the TC dish and removed by decanting to keep light exposure and washing procedure as short as possible. In the last step tilt dishes to remove all residual PBS with a pump. Complete removal of any liquid is required for equal crosslinking throughout the dish.

8. Crosslink at mild conditions using a 365 nm UV light source at 0.15 J/cm^3^.

***Note:*** Place TC dishes without lid as horizontally as possible on a tray of ice in the UV-crosslinker.

9. After crosslinking, add 20 mL of cold PBS onto TC dish, gently scrape cells into 50 mL tube, and spin down at 700xg for 5 minutes at 4°C.

***Note:*** Omit steps 9 and 10 when working with cells that are too adherent to be detached. Instead, lyse such cells in 600 μL Buffer A in the plate and continue with step 11.

10. Remove PBS from the cell pellet. Resuspend cell pellet in 3x pellet volume of Buffer A. Lyse cells for 10 minutes on ice.
11. Transfer cell lysate into microtube and add 1 μL of RNAse inhibitor (40 U/μL) and 10 μL of DNase I (10 U/μL) directly into cell lysate. Incubate for 5–10 min at 37°C.
12. Spin the lysate at 4°C for 20 minutes 16,000 xg and collect supernatant (SN) in a new tube. Take 1/10 of protein lysate as input sample and store at −80°C until step 15.
13. If targeting Myc-tagged exogenous protein: wash Anti-c Myc magnetic beads (50 μL per sample) on magnetic rack 3 times in Buffer A. Skip this step when targeting endogenous protein, in which case beads preparation instructions can be found in step 5.

***Note:*** Wash beads as mastermix rather than individual samples to limit variance of bead volume between samples. In the last wash, distribute beads into microtubes.

14. Remove Buffer A from beads and add cell lysate. Facilitate pull-down by gentle rotation at 4°C for 16 h.

#### Day 4

15. Place Buffer F at 20°C–25°C to dissolve precipitated SDS, and thaw input samples (stored at −80°C) on ice.
16. Collect magnetic beads on magnetic rack.

***Optional:*** Take 1/10 of unbound protein lysate fraction as flow through sample and continue their preparation like the preparation of the input samples.

17. Wash beads once with 900 μL of Buffer B, then 3 times with 900 μL of Buffer C.

***Optional:*** Take a 5% aliquot of bead slurry in Buffer C to assess pull-down efficiency by Western blotting. Replace Buffer C with Buffer G and cook samples for 5 min at 95°C. Then use SN with eluted protein for Western blotting.

18. To release protein-RNA complexes, fully replace Buffer C with 100 μL of Buffer F and incubate at 70°C for 10 minutes.
19. In the meantime, add equal amounts of Buffer D and F to “Input” and (optional) “Flow through” samples to the final volume of 200 μL. E.g. Input sample = 30 μL, add 85 μL of Buffer D and 85 μL Buffer F.
20. Transfer SN of pull-down (PD) samples on the magnetic rack into a new microtube, and add 100 μL of Buffer D.
21. To all samples (Pull-down, Input, optional Flow through) add 10 μL of proteinase K solution (20.3 mg/mL) and incubate for 30 minutes at 37°C. Subsequently add 200 μL of Buffer E to all samples and incubate for another 30 minutes on 37°C.
22. Add 400 μL of acidic phenol/chloroform and vortex vigorously.

**CRITICAL:** For personal safety perform these steps in a chemical hood.

23. Spin samples for 10 minutes at 16,000 xg at 4°C and transfer the aqueous phase (∼300 μL) into a new microtube.
24. For RNA precipitation, add.
  a. 0.5 μL of Glycoblue (15 mg/mL),
  b. 1/10 of the sample volume of 3 M sodium acetate.
  c. 1 mL of molecular biology grade 96 % EtOH.
25. Precipitate at −20°C for 16 h. Alternatively, precipitation can be performed for 1–2 h at −80°C.

#### Day 5

26. Centrifuge at 16,000 xg for 30 minutes at 4°C to collect RNA.
27. Wash pellet twice in ice-cold 80% EtOH.
28. After washing, remove all EtOH, air-dry the pellet for 5–10 minutes at 20°C–25°C.
29. Resuspend PD samples in 15–20 μL and Input and flow through samples in 200-300 μL RNase/DNase-free water.

#### Days 6–8

30. Remove DNA contamination: This step was performed by Bestehorn et al.^1^ but can be omitted, since DNase digest was already performed during cell lysis.
  a. Digest 5 μg of Input RNA in 1x DNase I buffer, supplemented with 1 μL of RNase inhibitor (40 U/μL) and 1 μL DNase I (10 U/μL) for 20 min at 37°C in an end volume of 50 μL H_2_O in nuclease-free water.
  b. After DNase digestion, dilute samples further in nuclease-free water to an end volume of 200 μL.
  c. For RNA isolation, add an equal amount of acidic phenol-chloroform (200 μL) to the sample, vortex the sample vigorously and spin down for 10 min at 16,000 xg at 4°C.
  d. Transfer the aqueous phase into a new microtube and precipitate RNA by sodium acetate and ethanol precipitation (→ see step 24).
  e. After precipitation at −20°C for 16 h repeat washing of RNA pellet as in steps 25 - 28 and resuspend input DNA digested samples in 50 μL of nuclease-free water.
31. Check RNA quality using Agilent RNA 6000 Nano Assay on an Agilent 2100 Bioanalyzer.

In Bestehorn et al.,^1^ steps 32 and 33 were carried out by the Next Generation Sequencing Facility at Vienna BioCenter Core Facilities (VBCF), member of the Vienna BioCenter (VBC), Austria.

32. Perform rRNA depletion and library preparation with a kit that accommodates strand-specificity.
33. Sequence with at least 5 million reads per sample (after processing & deduplication; the more the better) and preferably paired end.

### Read processing and alignment

**Timing: 1 day**

This step includes the processing of raw reads to obtain a bam file of aligned reads which is required for the subsequent custom analysis. Furthermore, we recommend creating coverage tracks to help with troubleshooting.

34. Perform read trimming to remove adapter, poly A and poly G sequences. We use trim-galore and Fastp. Run quality control (FastQC) before and after each step to monitor how these operations affect your reads.
35. Align reads to a reference genome (STAR), briefly check output SAM files and then convert them to BAM files including deduplication and sorting by name (Samtools).

**CRITICAL:** Sorting by name is required for counting with Htseq count for paired-end data.

36. For generation of coverage tracks, BAM files need to be sorted by position and indexed. Then, bamCoverage can be used for track generation. For stranded data, make sure to keep this information and generate separate tracks for each strand using the argument “--filterRNAstrand”.

module load deeptools

$bam = "<yourBAM>" **#your position sorted indexed BAM file**

bamCoverage --bam ${bam} \

 --outFileName ${file//.bam/.fwd.bw} --binSize 1 \

 --numberOfProcessors 16 --filterRNAstrand forward \

 --normalizeUsing CPM

bamCoverage --bam ${bam} \

 --outFileName ${file//.bam/.rev.bw} --binSize 1 \

 --numberOfProcessors 16 --filterRNAstrand reverse \

 --normalizeUsing CPM

## Key resources table

**Table:** 

REAGENT or RESOURCE	SOURCE	IDENTIFIER
**Chemicals, peptides, and recombinant proteins**		

Water	Fisher Chemical	Cat# W6-1
Pierce Anti-c-Myc magnetic beads	Thermo Scientific	Cat# 88843
Pierce Protein A/G magnetic beads	Thermo Scientific	Cat# 88803
4-thiouridine (4sU)	Sigma	Cat# T4509
RNasin ribonuclease inhibitor	Promega	Cat# N2511
Proteinase K	Roche	Cat# 3115828001
DNaseI buffer	Roche	Cat# 4716728001
DNaseI recombinant, RNase free	Roche	Cat# 4716728001
Acid-Phenol:Chloroform, pH 4.5 (with IAA, 125:24:1)	Thermo Scientific	Cat# AM9722
Lipopolysaccharides from *Escherichia coli* O55:B5 (LPS)	Sigma	Cat# L2637
Doxycycline hyclate	Sigma	Cat# D9891
TRIS hydrochloride (Tris-HCl)	Sigma	Cat# PHG0002
Sodium chloride (NaCl)	Sigma	Cat# S9888
Sodium dodecyl sulfate (SDS)	Sigma	Cat# 11667289001
NP-40 Surfact-Amps detergent solution	Thermo Scientific	Cat# 85124
Sodium deoxycholate monohydrate	Thermo Scientific	Cat# B20759.14
Magnesium chloride hexahydrate (MgCl_2_)	Sigma	Cat# M9272
Tween 20	Sigma	Cat# 11332465001
EDTA Disodium salt 2-hydrate for molecular biology	AppliChem	Cat# A2937.0500
Urea	AppliChem	Cat# A1049
Sodium acetate	Sigma	Cat# S8750
Ethanol absolute for HPLC	Sigma	Cat# 34852
cOmplete mini EDTA-free protease inhibitor cocktail	Sigma	Cat# 11836170001
Penicillin/Streptomycin	Sigma	Cat# P7794 / Cat# S9137
Fetal bovine serum (FBS)	Sigma	Cat# F7524
Dulbecco’s phosphate-buffered saline (PBS)	Sigma	Cat# D8537
DMEM – high glucose	Sigma	Cat# D5796
2-Mercaptoethanol	Sigma	Cat# M3148
Bromophenol blue	AppliChem	Cat# A3640
DL-Dithiothreitol (DTT)	Sigma	Cat# 43815
Invitrogen GlycoBlue	Invitrogen	Cat# AM9516

**Critical commercial assays**		

RNA 6000 Nano Assays	Agilent	Cat# 5067-1511
rRNA depletion kit Ribovanish	www.viennabiocenter.org/facilities	N/A
Ultra II Directional RNA Library Prep Kit	NEB	Cat# E7765

**Deposited data**		

Data this protocol were applied for in Bestehorn et al.^1^	Sequence Read Archive (SRA)	PRJNA1031176

**Experimental models: Cell lines**		

RAW ΔTTP: TTP-WT-3xMyc	Bestehorn et al.^1^	N/A

**Software and algorithms**		

Trim Galore (version 0.6.10)	Krueger F., Babraham Institute	RRID:SCR_014583
fastp (version 0.23.4)	Chen et al.^4^	https://github.com/OpenGene/fastp; RRID:SCR_016962
STAR (version 2.7.11a)	Dobin et al.^5^	RRID:SCR_004463
SAMTOOLS (version 1.18)	Li et al.^6^	RRID:SCR_002105
Deeptools bamCoverage (version 3.5.2)	Ramírez et al.^7^	https://www.htslib.org
FastQC (v.0.12.1)	Andrews^8^	https://www.bioinformatics.babraham.ac.uk/projects/fastqc/
htseq-count (version 2.0.4)	Anders et al.^9^	https://pypi.python.org/pypi/HTSeq; RRID:SCR_011867
Bedtools (version 2.30.0)	Quinlan and Hall^10^	https://bedtools.readthedocs.io/en/latest/content/overview.html; RRID:SCR_006646
R (version 4.4.1)	R Core Team^11^	https://www.r-project.org/; RRID:SCR_001905
tidyverse (version 2.0.0)	Wickham et al.^12^	https://www.tidyverse.org/packages/; RRID:SCR_019186

## Materials and equipment

**Table:** Buffer A: RIPA lysis buffer

Reagent	Final concentration
Tris-HCl pH 7.4	50 mM
NaCl	100 mM
NP-40	0.1%
SDS	0.1%
sodium deoxycholate	0.5%
cOmplete protease inhibitor	1x

Prepare freshly, sterile filter, store at 4°C until use.

**CRITICAL:** NP-40 can cause skin corrosion and eye damage; it is toxic to aquatic environment. Do not handle without protective gloves/ protective clothing, and eye protection. Avoid release to the environment. SDS is toxic and a flammable solid. Wear protective gloves, clothing and eye protection. Avoid breathing dust/fumes and avoid release to the environment. Sodium deoxycholate can cause acute oral toxicity and may cause respiratory irritation. Handle with protective gloves and clothing in toxic fume hood to avoid breathing dust/fume.

**Table:** Buffer B: High salt buffer

Reagent	Final concentration
Tris-HCl pH 7.4	50 mM
NaCl	1 M
EDTA	1 mM
NP-40	1%
SDS	0.1%
sodium deoxycholate	0.5%
cOmplete protease inhibitor	1x

Prepare freshly, sterile filter, store at 4°C until use.

**CRITICAL:** NP-40 can cause skin injury and eye damage. Do not handle without protective gloves/ protective clothing and eye protection. Avoid release to the environment. SDS is toxic and a flammable solid. Wear protective gloves, clothing and eye protection. Avoid breathing dust/fumes and avoid release to the environment. Sodium deoxycholate is harmful if swallowed and may cause respiratory irritation. Handle with protective gloves and clothing in toxic fume hood to avoid breathing dust/fume.

**Table:** Buffer C: Wash buffer

Reagent	Final concentration
Tris-HCl pH 7.4	20 mM
MgCl_2_	10 mM
TWEEN 20	0.2%
cOmplete protease inhibitor	1x

Prepare freshly, sterile filter, store at 4°C until use.

**Table:** Buffer D: Proteinase K adjustment buffer

Reagent	Final concentration
Tris-HCl pH 7.4	100 mM
NaCl	50 mM
EDTA	10 mM

Prepare freshly, sterile filter, store at 20°C–25°C until use.

**Table:** Buffer E: Proteinase K urea buffer

Reagent	Final concentration
Tris-HCl pH 7.4	100 mM
NaCl	50 mM
EDTA	10 mM
Urea	7M

Prepare freshly, sterile filter, store at 20°C–25°C until use.

**Table:** Buffer F: SDS-elution buffer

Reagent	Final concentration
Tris-HCl pH 6.5	50 mM
DTT	100 mM
SDS	2%

Prepare freshly, sterile filter, store at 4°C until use.

***Note:*** SDS will precipitate at 4°C, and the buffer will look turbid.

**CRITICAL:** DTT is harmful if swallowed, causes skin irritation and causes serious eye damage. While handling, wear protective gloves, clothing and eye protection. SDS is toxic and a flammable solid. Wear protective gloves, clothing and eye protection. Avoid breathing dust/fumes and avoid release to the environment.

**Table:** Buffer G: SDS loading dye

Reagent	Final concentration
Tris-HCl pH 6.5	0.25 M
SDS	10%
Glycerol	50%
2-Mercaptoethanol	20%
bromophenol blue	100 mM

Prepare freshly, sterile filter, store at 4°C until use.

***Note:*** SDS will precipitate at 4°C, and the buffer will look turbid.

**CRITICAL:** SDS is toxic and a flammable solid. Wear protective gloves, clothing and eye protection. Avoid breathing dust/fumes and avoid release to the environment. 2-Mercaptoethanol is toxic if swallowed or inhaled. It causes skin irritation and serious eye damage. Work in toxic chemical fume hood with protective clothing, gloves and eye protection.

## Step-by-step method details

The following steps detail the computational part of this analysis to obtain PD-enriched genes and calculate the ratio of intronic to exonic coverage (InEx). File names in the code snippets do not include directories as they are only placeholders for your preferred file name and location. Code for steps 1 - 7 is bash/awk; from step 8 onwards this protocol uses R code.

### Adaption of the reference genome

**Timing: 1 day**

In this step, we adapt a reference genome to contain only unambiguous exon and intron annotations for each gene.

**Figure 1:**
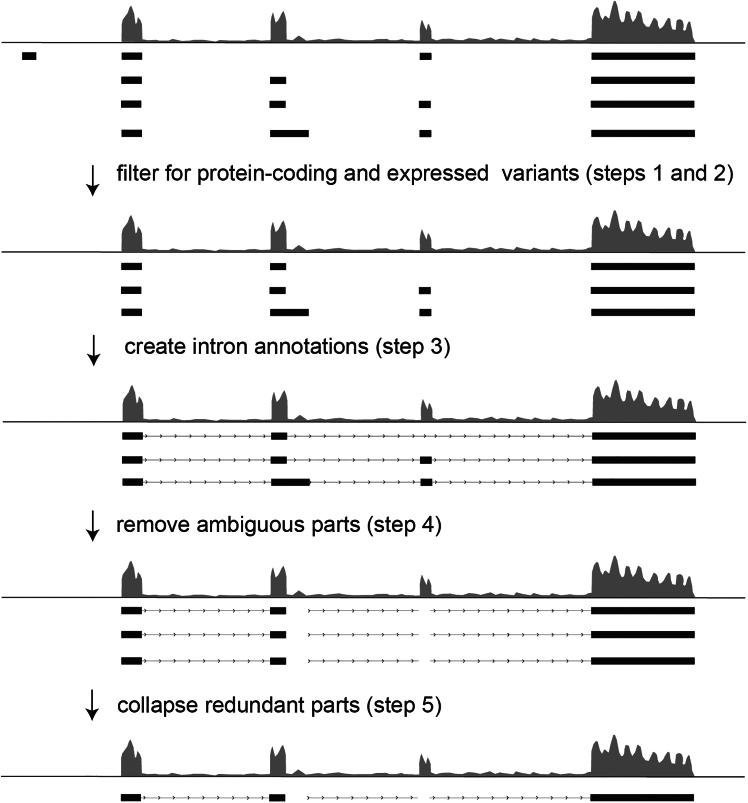
Flowchart of reference genome processing (steps 1–5) A schematic track of RNA-seq coverage is depicted in gray with annotations in black below. Exons are shown as boxes, introns as lines and arrows indicate the orientation of the gene. Prior to processing, reference genomes include exon annotations for each known transcript variant of each gene. In this scheme, one transcript variant includes a more upstream exon which does not have RNA-seq fragment coverage, and some exons and introns of different transcript variants partially overlap. Steps 1 and 2 of the reference genome processing filter for protein-coding and fully expressed variants, thereby getting rid of the variant with the more upstream exon. In step 3, intron annotations are added to the reference genome, so that in step 4, any overlapping parts of exon and intron annotations can be removed. This leaves several redundant annotations of unambiguous exons and introns for each gene, which are finally collapsed into a non-redundant and unambiguous reference genome in step 5.

***Note:*** Annotations of intronic and exonic sequences are often ambiguous due to differential splicing. Depending on splice variants, the same sequence can remain in the mature mRNA or be spliced out. Hence, to precisely quantitate intronic and exonic fragments bound to the RBP it is important to count only fragments mapped to unambiguously annotated sequences. This exact counting ensures that intronic fragments, i.e. low coverage RNA regions, are not contaminated with the much more abundant exonic RNA regions. Therefore, the reference genome used for this counting must contain only unambiguous intron and exon annotations, which we accomplish in this step as illustrated in Figure 1. The following code is developed with an Ensembl reference genome and may have to be adapted for genomes from other sources.

1. When working with an RBP that binds protein-coding transcripts: Filter your Ensembl GTF file for exon annotations of protein coding transcript variants.
  ***Note:*** Omit this step in case you are interested in an RBP that binds non-coding RNAs. Since total RNA-seq includes highly abundant transcripts that may overlap with non-coding RNAs, we do not recommend filtering for only annotations of e.g. long non-coding RNAs (lncRNAs).
  a. Perform the filtering.
    awk -F '\t' 'BEGIN {OFS="\t"}
    ($3=="exon" &&
    ($9 ∼ /"protein_coding"/ ||
     $9 ∼ /"protein_coding_LoF"/))' \
    genome.gtf > 1_protein.coding.exons.gtf
  b. Browse through 1_protein.coding.exons.gtf and make sure that there are only annotations for protein coding transcripts.
2. Filter for transcript variants which have at least one fragment overlapping with each exon.
  This way, transcript variants which are not expressed in any of the samples are excluded from the analysis. Troubleshooting.
  a. For each of your samples, count the fragments overlapping with each exon annotation in 1_protein.coding.exons.gtf using htseq count. Subsequently, extract the exon IDs for exons that have counts > 0 for each file.
    ***Note:*** This step will give you one TSV file for each sample and can be sped up by performing it as an array job if you have adequate computational resources.
    **CRITICAL:** The argument “--nonunique all” is required for fragments that overlap with more than one annotation to be counted for each of them. This is necessary when counting for individual exons as multiple transcripts for the same gene usually include the same exon.
    bam_files=∗.bam
    for bam in $bam_files; do
     out_tsv= 2a_${bam//.bam/.exoncounts.tsv}
     out_csv= 2a_${bam//.bam/.usedexons.csv}
     # **count fragments overlapping for each exon**
     htseq-count -f bam $bam \
     -t exon -i exon_id -m union --nonunique all \
      --order=name -s reverse \
      1_protein.coding.exons.gtf \
     -c ${out_tsv}
     # **now get exon IDs with counts > 0**
     awk -F'\t' '$1 !∼ /ˆ__/ && $2 > 0 {
      print $1
     }' ${out_tsv} > ${out_csv}
    done
  b. Make a list of exons that have counts > 0 in any of the samples and filter your GTF file for those exons.
    # **make list of used exons**
    cat ∗.usedexons.csv | sort -u > 2b_usedexons.txt
    **# filter the GTF file for lines with these exon ids**
    grep -Fwf 2b_usedexons.txt \
     1_protein.coding.exons.gtf > 2b_used.exons.gtf
  c. Sort both 1_protein.coding.exons.gtf and 2b_used.exons.gtf by transcript ID and chromosomal start position.
    # **sorting annotations by transcript ID and position**
    **# original annotations file**
    awk -F'\t' 'BEGIN { OFS=FS }
      # **adding a column with transcript ID**
     { split($9, a, "[ ;]");
      #split column 9 by ; and extract transcript ID
      for (i = 1; i <= length(a); i++) {
     if (a[i] == "transcript_id") {
     transcript_ID = a[i+1];
     break;
     }
      }
      print $0, transcript_ID
     #**print original line plus transcript ID**
     }
    ' 1_protein.coding.exons.gtf | \
     sort -t$'\t' -k10,10 -k4,4n > 2c_exons.sorted.gtf
    # **used exons file**
    awk -F'\t' 'BEGIN { OFS=FS }
     **# adding a column with transcript ID**
     { split($9, a, "[ ;]");
     **#split column 9 by ; and extract transcript ID**
     for (i = 1; i <= length(a); i++) {
      if (a[i] == "transcript_id") {
      transcript_ID = a[i+1];
      break;
      }
     }
     print $0, transcript_ID
      **#print original line plus transcript ID**
     }
    ' 2b_used.exons.gtf | \
     sort -t$'\t' -k10,10 -k4,4n > \
     2c_used.exons.sorted.gtf
  d. For both 2c_used.exons.sorted.gtf and 2c_exons.sorted.gtf, count the exons per transcript ID and put the output in CSV files.
    # **for used exons**
    awk 'BEGIN {FS="\t"; OFS=","; transcript_ID = ""}
     {
     current_transcript_ID = $10
     if (current_transcript_ID != transcript_ID){
      # **when encountering a new transcript ID**
      if (transcript_ID != "") {
     **# and it’s not the first line, print the exon count for the previous transcript ID**
     print transcript_ID, exon_count
      } else {
     **# if it is the first print a header**
     print "transcript_ID", "exon_count"
      }
      # **after having printed the previous define this new transcript ID as the one to be counted and set the count to 1**
      transcript_ID = current_transcript_ID
      exon_count= 1
     } else {
     **# if it’s not a new ID: add 1 to the count**
     exon_count++
     }
    }' 2c_used.exons.sorted.gtf > 2d_used.exons.count.csv
    **# same for all exons**
    awk 'BEGIN {FS="\t"; OFS=","; transcript_ID = ""}
     {
     current_transcript_ID = $10
     if (current_transcript_ID != transcript_ID){
     if (transcript_ID != "") {
     print transcript_ID, exon_count
     } else {
     print "transcript_ID", "exon_count"
     }
     transcript_ID = current_transcript_ID
     exon_count= 1
     } else {
     exon_count++
     }
    }' 2c_exons.sorted.gtf > 2d_exons.count.csv
  e. Compare the exon counts in the two CSV files, if the count is the same, put the transcript ID in a TXT file.
    awk -F, '
     **# saving exon counts for each transcript ID from first file**
     FNR==NR {exon[$1]=$2; next}
     **# if the count in second file is the same as in first file print transcript ID to text file**
     FNR>1 {if (exon[$1]==$2) print $1}
    ' 2d_exons.count.csv 2d_used.exons.count.csv > \
     2e_used.TVs.txt
  f. Filter 2c_exons.sorted.gtf for annotations with transcript IDs in 2e_used.TVs.txt.
    ***Note:*** The output file 2f_exp.TVs.exons.gtf now contains exon annotations for protein-coding transcript variants showing read coverage in all exons.
    grep -Fwf 2e_used.TVs.txt \
     2c_exons.sorted.gtf > 2f_exp.TVs.exons.gtf
  g. For a few randomly selected genes, check the exon annotations in 2f_exp.TVs.exons.gtf. Verify that all exons of a transcript variant are included. In addition, use the coverage tracks (see Read processing and alignment) to verify that transcript variants without coverage were excluded.
3. Create intron annotations, defined as the sequences between exons in each remaining transcript variant and save this as a separate GTF file.
  ***Note:*** You now have one GTF file with exon and one with intron annotations.
  **CRITICAL:** It is important to have the exon annotation file sorted first by transcript ID and then by genomic position. Since the output file from step 2f is already sorted this way, the sorting does not have to be re-done but consider this in case you adapt any of the above codes.
  a. Create intron annotations file.
    awk 'BEGIN {FS="\t"; OFS="\t"}
     {
     current_transcript_ID = $10;
     if (current_transcript_ID != transcript_ID) {
      **# when encountering a new TV, no intron will be saved (this is only the first exon) -> only save the end position of this exon + 1 as the start position of the next intron**
      transcript_ID = current_transcript_ID;
      prev_end = $5+1
     } else {
      # **if this is not a new transcript variant**
      if (prev_end && $4 > prev_end) {
      **# and there has been a previous exon and the current one is further downstream than the previous one (which should be the case due to the sorting) -> print an entry for the intron defined as the sequence between the two exons**
     print $1, "calculated", "intron", prev_end, $4-1, $6, $7, $8, $9, transcript_ID
      }
     prev_end = $5+1;
     }
     }' 2f_exp.TVs.exons.gtf > 3_exp.TVs.introns.gtf
  b. For a few randomly selected transcripts, manually check if intron annotations are flanked by exon annotations on both sides and there is an intron annotation between all exons of a transcript.
4. Remove ambiguous parts (i.e. annotated as exon in one transcript variant and intron in another) using bedtools subtract.
  a. Convert your GTF files to BED files and sort them by position.
    **# exon annotations**
    awk -F'\t' 'BEGIN { OFS="\t" }
    {
     split($9, a, "[ ;]");
     for (i = 1; i <= length(a); i++) {
     if (a[i] == "gene_id") {
     gene_id = a[i+1];
     break;
     }
     }
     print $1, $4-1, $5, gene_id, ".", $7, "."
    }' 2f_exp.TVs.exons.gtf | \
     sort -k1,1 -k2,2n > 4_exp.TVs.exons.bed
    # **intron annotations**
    awk -F'\t' 'BEGIN { OFS="\t" }
    {
     split($9, a, "[ ;]");
     for (i = 1; i <= length(a); i++) {
     if (a[i] == "gene_id") {
     gene_id = a[i+1];
     break;
     }
     }
     print $1, $4-1, $5, gene_id, ".", $7, "."
    }' 3_exp.TVs.introns.gtf | \
     sort -k1,1 -k2,2n > 4_exp.TVs.introns.bed
  b. Run bedtools subtract to get two BED files with only unambiguous exon or only unambiguous intron annotations.
    **# remove overlapping parts from exon annotations**
    bedtools subtract -a 4_exp.TVs.exons.bed \
      -b 4_exp.TVs.introns.bed -s > 4_unamb.exons.bed
    **# remove overlapping parts from intron annotations**
    bedtools subtract -a 4_exp.TVs.introns.bed \
      -b 4_exp.TVs.exons.bed -s > 4_unamb.introns.bed
  c. Count the number of annotations (i.e. lines) in input and output files. For a few randomly selected genes, manually ensure the correct removal of ambiguous sequence parts.
    ***Note:*** For the intron file, the number of annotations should be higher in output while for the exon file this number should be lower. This is because the removal of an ambiguity region (i.e. an alternatively spliced exon) results in the loss of the exon and splits the intron into two parts, thereby increasing the number of intron annotations. Conversions between BED and GTF should not affect the line number.
5. Collapse the exon or intron annotations, remove any annotations where genes overlap and merge the exon and intron files into one genome file containing unambiguous exon and intron annotations for each gene.
  a. Collapse the exon or intron annotations using bedtools merge.
    **CRITICAL:** Input BED files must be sorted by position. Do this despite the sorting carried out in the beginning of step 4a as the annotations have changed since then.
    ***Note:*** The output will be two BED files with non-redundant intron and exon annotations, respectively. These files will also show gene IDs of genes that are included in each merged annotation. In case of overlapping genes, this column will list all gene IDs separated by a comma.
    **# sort the BED files by position before collapsing**
    sort -k1,1 -k2,2n 4_unamb.exons.bed > 5_unamb.ex.sort.bed
    sort -k1,1 -k2,2n 4_unamb.introns.bed > 5_unamb.in.sort.bed
    **# collapse each BED file**
    bedtools merge -s -c 4,6,7 -o distinct,distinct,distinct \
      -i 5_unamb.ex.sort.bed > 5_col.ex.bed
    bedtools merge -s -c 4,6,7 -o distinct,distinct,distinct \
      -i 5_unamb.in.sort.bed > 5_col.in.bed
  b. Remove regions assigned to more than one gene making use of the comma-separated format and convert back to GTF.
    # **filter out lines from > 1 gene + convert back to GTF file:**
    awk -F'\t' -v OFS='\t' '!($4 ∼ /,/) {
     print $1, ".", "exon", $2 + 1, $3, ".", $5, ".",
     "gene_id "$4
    }' 5_col.ex.bed > 5_col.ex.gtf
    awk -F'\t' -v OFS='\t' '!($4 ∼ /,/) {
     print $1, ".", "intron", $2 + 1, $3, ".", $5, ".",
     "gene_id "$4 }'
    5_col.in.bed > 5_col.in.gtf
  c. Combine the two annotations files into one. You now have a customized GTF file with non-redundant and unambiguous intron and exon annotations.
    #**merge into one GTF file**
    cat 5_col.ex.gtf 5_col.in.gtf > 5_annotations.gtf
  d. Count the number of annotations (i.e. lines) in all files. For a few randomly selected genes, look at all known transcript variants and your fragment coverage tracks to see that (i) there are no ambiguous annotations left, and (ii) all annotations show coverage in the tracks.
    ***Note:*** The number of annotations should decrease for both intron and exon annotation in the collapsing step. Conversion to GTF by itself does not change the number of annotations. However, since we remove regions assigned to more than one gene in the same step, the number of annotations will decrease, albeit to a much smaller extent (in the range of hundreds). The number of annotations in the combined file should be the same as the sum of the individual ones.
6. Generate a file that contains exon/intron lengths for each gene, derived from the unambiguous collapsed intron and exon annotations.

***Note:*** Counts will be normalized by exon and intron lengths in step 10 for which this file will be needed.

**# sort by geneID and then in positional order**

sort -t $'\t' -k9 -k4n 5_annotations.gtf > \

 6_sorted.annotations.gtf

**# determine intron and exon length**

awk 'BEGIN {FS="\t"; OFS=","; gene_id = ""}

 { current_gene_id = $9

 #**strip ‘geneID "’ and ‘"’ to keep just the ID itself**

 gsub(/ˆgene_id "/, "", current_gene_id);

 gsub(/"$/, "", current_gene_id);

 # **when transitioning to next gene, print the values**

 if (current_gene_id != gene_id) {

 if (gene_id != "") {

 print gene_id, in_length, ex_length

 } else { **# print headers in first row**

 print "gene_id","in_length","ex_length"

 }

 # **calculate length of the first annotation of a gene**

  gene_id = current_gene_id

 in_length= 0

 ex_length= 0

 if ($3 == "intron") {

  in_length=$5 - $4 +1

 } else if ($3 == "exon") {

  ex_length=$5 - $4 +1

 }

 } else if ($3 == "intron") {

 **# while staying within the same gene: add**

   **exon/intron length to previous values**

 in_start=$4

 in_end=$5

 in_length=in_length + in_end - in_start + 1

 } else if ($3 == "exon") {

 ex_start=$4

 ex_end=$5

 ex_length=ex_length + ex_end - ex_start + 1

 }

 }

 END {**# print last gene**

  if (gene_id != "") {

 print gene_id, in_length, ex_length

  }

 }' 6_sorted.annotations.gtf > 6_intron_exon_length.csv

### Fragment counting

**Timing: 30 min working time, 1.5 h run time per sample**

In this step, we quantitate exonic and intronic RNA-seq fragments using the adapted reference genome.

***Note:*** Intronic fragments were defined as fragments overlapping with introns or exon-intron boundaries while exonic fragments were fragments overlapping with exons or exon-exon-junction or exon-intron boundaries. This allows us to estimate the proportion of unspliced transcripts (in the pool of unspliced and spliced RNA) through the ratio of intronic and exonic counts (InEx).

In addition, we need to calculate the library size for normalization in step 9. The library size is the sum of all fragments overlapping with either exon or intron annotations, exon-exon junctions or exon-intron boundaries of all genes.

Both intronic and exonic counts, as defined above, include exon-intron boundary overlapping fragments. Hence, if we were to sum them up to obtain the library size, we would count these fragments twice. Therefore, we separately count exon and exon-exon junction overlapping fragments and sum them with the intron and exon-intron boundary overlapping fragments for the determination of library size.

7. Count intronic, exonic and strictly exonic fragments:
  a. Count fragments overlapping introns and exon-intron boundaries using the arguments “-t intron” and “-m union”.
    htseq-count -f bam ${file} \
     -t intron -i gene_id -m union --nonunique all \
     --order=name -s reverse \
     5_annotations.gtf \
     -c ^1^7a_${file//.bam/.intron.tsv}
  b. Count fragments overlapping exons, exon-exon junctions and exon-intron boundaries using the arguments “-t exon” and “-m union”.
    htseq-count -f bam ${file} \
     -t exon -i gene_id -m union --nonunique all \
     --order=name -s reverse \
     5_annotations.gtf \
     -c ^1^7b_${file//.bam/.exon.tsv}
  c. Count fragments overlapping exons and exon-exon junctions using the arguments “-t exon” and “-m intersection-strict”.
    htseq-count -f bam ${file} \
     -t exon -i gene_id -m intersection-strict \
     --nonunique all --order=name -s reverse \
     5_annotations.gtf \
     -c ^1^7c_${file//.bam/.exon.strict.tsv}
  d. Check your output files from steps 7a-c. Pay attention to whether your gene counts follow expected tendencies (highly expressed genes and genes you expect to be pulled down have high counts in Input and PD samples, respectively).
    ***Note:*** This operation will leave you with one count file per sample and per category. Count files should have two columns, geneID and counts. Each row in these files shows the counts of fragments as defined above for a gene. Troubleshooting

### Normalization

**Timing: 2 h**

In this step we make counts comparable between samples as well as between the intronic and exonic counts within a sample.

***Note:*** To make counts comparable between samples, we need to normalize for library size.

For the comparison of intronic and exonic counts within a sample, we need to consider that the counts originate from different gene regions, i.e. introns and exons which can substantially differ in their length. Therefore, we need to normalize the intronic and exonic counts by intron and exon lengths, respectively.

8. Create raw count tables from the above created count files and store them in a list of raw count tables (data.raw).

**# load necessary packages**

library(tidyverse)

# **function for loading TSV files and renaming their count column to the sample name**

>read.in<-function(element){

> new.name<-str_replace(element,"\\.tsv$","")%>%

> str_replace("ˆ7[abc]_","")

> read_tsv(element, col_names=c("gene",new.name))

>}

# **function to list all TSV files for intronic, exonic or exonic (strictly) counts and make a raw count table each from them**

>raw.read<-function(suffix){

> raw.count.df<-list.files(pattern=suffix)%>%

> lapply(read.in)%>%

> purrr::reduce(full_join, by = "gene")%>%

> as.data.frame()%>%

> mutate(gene=str_replace(gene, "\\.[0-9]+$", ""))%>%

> filter(!grepl("ˆ__", gene))%>%

> column_to_rownames("gene")

> return(raw.count.df)

>}

# **apply the functions to make your list of raw count dfs**

>setwd("yourpath") **#yourpath = path to directory with TSV files**

#**suffixes that distinguish files for intronic, exonic and strictly exonic counts**

>suffixes<-c("intron.tsv", "exon.tsv", "exon.strict.tsv")

#**create df list**

>data.raw<-lapply(suffixes,raw.read)

#**name list elements to intuitively access dfs**

>names(data.raw)<-c("intron","exon","exon.strict")

9. Perform count per million (CPM) library size normalization.
  a. Calculate library size. For each sample, sum up all intronic and strictly exonic counts.
    #library size = intronic (union) plus exonic (intersection-strict) counts
    >lib.size<-colSums(data.raw[["exon.strict"]])+
    > colSums(data.raw[["intron"]])
  b. Calculate CPM values and store the output in a list of CPM normalized count tables.
    **# library size normalization**
    >norm.CPM<- function(df){
    > out.df<-sweep(df, 2, lib.size / 1e6, FUN = "/")
     **# 2 defines the calculation per column**
    > return(out.df)
    >}
    >data.CPM <- lapply(data.raw, norm.CPM)
  c. Manually check the calculation in the first rows. Ensure the division by library size happened per column and not repetitively per row.
10. Normalize to intron and exon lengths (per kb) and calculate the means for each condition.
  ***Note:*** The file containing the intron and exon lengths (6_intron_exon_length.csv) is generated in step 6.
  a. Perform the calculation.
    #**importing exon intron length information**
    >exin.length<-read.csv("6_intron_exon_length.csv")%>%
    > mutate(gene_id=str_replace(gene_id, "\\.[0-9]+$", ""))%>%
     **# removing version number from Ensembl gene IDs**
    > column_to_rownames("gene_id")%>%
    > subset(in_length!=0) **#removing single exon genes**
    # **add the intron and exon length columns to the library size normalized count tables**
    >data.CPM.lengths<-lapply(data.CPM, merge, y=exin.length,
    >  by="row.names")%>%
    > lapply(as.data.frame)
    # **divide all sample columns (in this case all starting with PD or Input) by either intron or exon length**
    # **function for common operations: removing length columns and adding condition average columns**
    >TPM.table.clean.up<-function(df){
    > df<-df%>%
    > select(-c(gene_length,in_length,ex_length))%>%
    > rowwise()%>%
    > mutate(mean_PD=mean(c_across(contains("PD")), na.rm=T),
    >  mean_Input=mean(c_across(contains("Input")),
    >  na.rm=T))%>%
    > ungroup()
    > return(df)
    >}
    >data.TPM<-list()
    >data.TPM[["intron"]]<-data.CPM.lengths[["intron"]]%>%
    > mutate(across(contains("PD_") | contains("Input_"),
    >  .fns = ∼ . / in_length ∗ 1000))%>%
    > TPM.table.clean.up()
    >data.TPM[["exon"]]<-data.CPM.lengths[["exon"]]%>%
    > mutate(across(contains("PD_") | contains("Input_"),
    >  .fns = ∼ . / ex_length ∗ 1000))%>%
    > TPM.table.clean.up()
  b. Manually check the calculation in the first rows.

### Enrichment analysis

**Timing: 1.5 h**

In this step, we perform a basic enrichment analysis on TPM normalized counts.

***Note:*** Targets of the RBP are identified by their enrichment in the PD fraction over Input. Since most genes are depleted in the PD fraction and few genes are strongly enriched, the assumption of a common scale factor that DESeq2 depends on^13^ is not met for this data.

11. Reduce false positives in enrichment analysis by setting a cutoff.
  ***Note:*** These false positives would originate from log-transformation-caused spreading of counts of lowly expressed genes and the concomitant introduction of noise in step 12. Therefore, filter for genes whose counts are above the mean in PD. Troubleshooting.
  a. Perform calculation and filtering.
    ***Optional:*** Save your filtered TPM count tables as intermediate files.
    # **calculate mean overall TPM in PD as cutoff value**
    >PD.mean<-mean(data.TPM[["intron"]]$mean_PD)
    **# remove genes where the mean exonic or intronic counts of PD samples is below cutoff**
    >data.TPM.cutoff<-data.TPM%>%
    > lapply(filter, mean_PD>PD.mean)%>%
    > lapply(column_to_rownames,"Row.names")
  b. Check if the filtering worked.
    ***Note:*** The number of rows should decrease and genes with low PD counts should be removed.
12. Log-transform normalized counts.

***Note:*** It is common practice to add a pseudocount to the normalized count value to avoid undefined values for not expressed genes here. However, genes with 0 counts were not of interest for this analysis and most likely already filtered out in step 11. Any remaining genes with nonfinite values after log-transformation are removed here.

**# function for removal of genes with nonfinite values (-Inf)**

>all.is.finite<-function(df){

> df<-df%>%

> filter_all(all_vars(is.finite(.)))

> return(df)

>}

**# log-transformation**

>data.log<-lapply(data.TPM.cutoff, log)%>%

> lapply(all.is.finite) **#removing rows where log-transf. gave -Inf**

>names(data.log)<-c("intron","exon")

13. Perform t-test and correct p-values (Benjamini-Hochberg correction).
  ***Note:*** We use t-test which expects normally distributed data, since RNA-seq data were shown to follow a log-normal distribution,^14^ i.e. after the log-transformation in step 12, your data should follow a normal distribution. Alternatively, Mann-Whitney U test can be used if it is not sure that the data are normally distributed.
  **CRITICAL:** Ensure t-test is performed per row using “rowwise()” and correction is done for all tests combined using “ungroup()”.
  a. Perform testing and correction.
    # **function for t-test and correction per df**
    >t.test.per.df<-function(df){
    > df<-df%>%
    > rownames_to_column("gene")%>%
    > rowwise() %>% **#important to run t.test per gene!**
    > mutate(p_val = t.test(c_across(contains("PD_")),
    >   c_across(contains("Input_")),
    >   alternative = "two.sided")$p.value)%>%
    > ungroup() %>%
     **#important to run correction across all tests!**
    > mutate(p_adj = p.adjust(p_val, method = "BH"))%>%
    > column_to_rownames("gene")
    > return(df)
    >}
    **# run t-test and p-value correction**
    >data.ttest<-lapply(data.log, t.test.per.df)
  b. Check your p-values and adjusted p-values.
    ***Note:*** The p-value should be different for each row (performed per gene, not over all genes) and the adjusted p-value should be higher than p-value. If this is not the case, the correction was not successful. This happens if the row-wise grouping is not removed before correction.
14. Calculate a log-Fold-Change (logFC) and add a classification for enriched genes.
  a. Perform the calculations.
    **# calculate a logFC:**
    >data.DE<-lapply(data.ttest, function(df){
    >  df<-df%>% mutate(logFC=mean_PD-mean_Input)
    >  return(df)
    >  })%>%
    > lapply(function(df){
    >  df<-df%>%
    > mutate("enriched"=case_when(
    >   p_adj <= 0.05 & logFC > 0 ∼ "enriched genes",
    >   T ∼ "not enriched genes"))
    >  return(df)
    >  })
  b. Manually check if your logFC values and the classification of enriched genes make sense.
15. Extract lists of significantly enriched genes in exonic and intronic counts and combine into one list.

***Optional:*** Save enriched.genes as a csv file.

**# individual lists for enriched genes in intronic or exonic counts**

>enriched.genes.lists<-lapply(data.DE, function(df){

>  df<-df%>%filter(p_adj <= 0.05 & logFC > 0)

>  return(df)

>  })%>%

>  lapply(rownames)

**# combined list**

>enriched.genes<-union(enriched.genes.lists$intron,

>   enriched.genes.lists$exon)

### InEx ratio

**Timing: 30 min**

In this step, we calculate the InEx ratio from the normalized intronic and exonic counts.

***Note:*** The comparison of InEx ratios in PD versus Input is needed to reveal the binding preference of your RBP for pre-mRNA of mature mRNA.

The InEx ratio can be higher than 1 in both Input and PD samples. This can happen due to inhomogeneity in sequencing coverage (that is intrinsic to RNA-seq) and/or annotation inaccuracy (see limitations). However, in the PD samples, a ratio higher than 1 for a given gene may indicate that the enriched RNA comprised a large amount of spliced out intron bound to the RBP.

**Figure 2:**
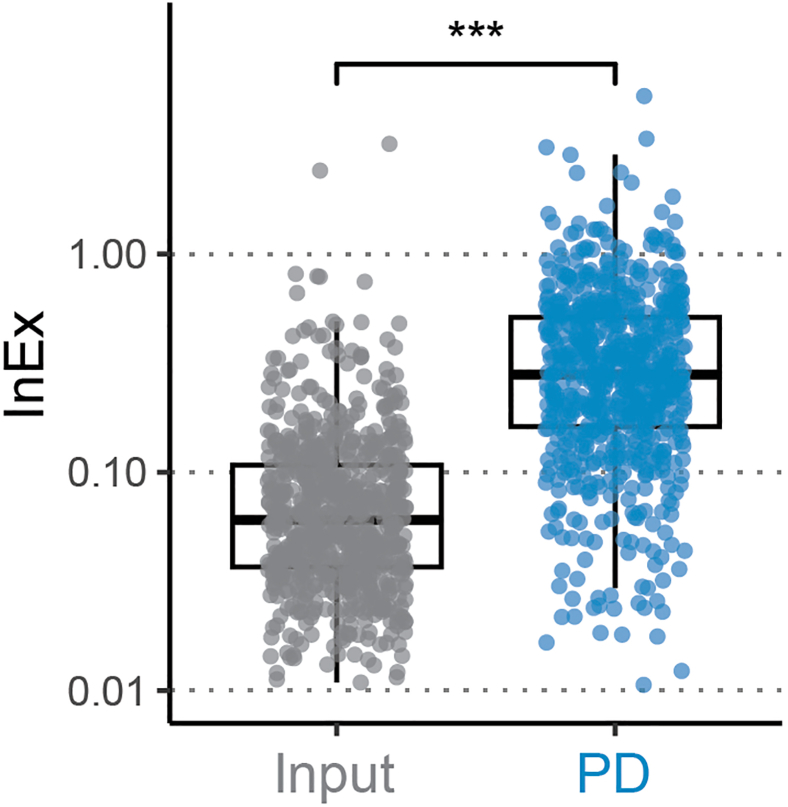
TTP prefers binding to pre-mRNA Mean InEx for each TTP pull-down (PD)-enriched gene in Input and PD is depicted together with boxplots indicating the quantiles of either group. The mean InEx is significantly increased by the PD (t-test, ∗∗∗ = p<0.001), revealing a higher percentage of unspliced RNA in the TTP-bound fraction. The conclusion is that TTP binds preferentially pre-mRNA.

16. Calculate InEx for enriched genes separately for each replicate.
  **CRITICAL:** Mean calculation must be conducted row-wise, hence grouping before this operation with the command “rowwise()” is required.
  a. Perform the calculations.
    # **calculate the InEx for enriched genes**
    >InEx<-merge(data.TPM.cutoff[["intron"]],
    >  data.TPM.cutoff[["exon"]],
    >  by="row.names",
    >  suffixes=c("_intron","_exon"))%>%
    > subset(Row.names %in% enriched.genes)%>%
      **#filtering for enriched genes**
    > select(-c(starts_with("mean")))%>%
    > pivot_longer(-Row.names, names_to = "sample",
    >   values_to = "TPM")%>%
    > mutate( **# this could be substituted by separate() if sample**
      **naming follows a pattern such as**
      **Input/PD_replicate_intron/exon**
    > "condition"=case_when( #extracting Input/PD
    >  grepl("Input",sample)∼"Input",
    >  grepl("PD",sample)∼"PD",
    >  T∼"inconsistent Input/PD naming!"),
    > "intron.exon"=case_when( #extracting intron/exon
    >  grepl("intron",sample)∼"intron",
    >  grepl("exon",sample)∼"exon",
    >  T∼"inconsistent intron/exon naming!"),
    > "replicate"=str_remove(sample, "ˆ(Input|PD)_")%>%
    >  str_remove("_(intron|exon)$"))%>%
     **# separate TPM columns for exon and intron**
    > pivot_wider(id_cols = c(Row.names, condition, replicate),
    >  names_from = intron.exon,values_from = TPM,
    >  names_glue = "TPM_{intron.exon}")%>%
     **# calculate InEx**
    > mutate("InEx"=TPM_intron/TPM_exon)%>%
    > select(-c(TPM_intron,TPM_exon))%>%
     # all replicates of either PD or Input per row
    > pivot_wider(id_cols = c(Row.names, condition),
    >  names_from = replicate,values_from = InEx,
    >  names_glue = "InEx_{replicate}")%>%
    > rowwise()%>%
     # **calculate mean InEx for each gene within condition**
    > mutate(mean_InEx=mean(c_across(starts_with("InEx"))))%>%
    > ungroup()
  b. Manually check a few lines to see if the calculations of the InEx and its means in Input and PD are correct.
    ***Note:*** If means are the same for all genes, mean calculation was not performed row-wise. This should be corrected using “rowwise()”
17. Compare InEx of genes of interest between the two experimental conditions, using e.g. histograms or boxplots of the mean values for PD-enriched genes (Figure 2) or boxplots of replicate values of single genes, all of which can be found in Bestehorn et al.^1^

## Expected outcomes

One result of the workflow above are TPM normalized counts, which in turn can be used for enrichment analysis as also described here, but also for example for regression analysis or analysis of covariance as shown in Bestehorn et al.^1^ Enrichment analysis determines genes whose RNA is more abundant in PD samples as opposed to Input samples, i.e. genes bound by the RBPs of interest. This mode of detection of RBP-targets is applied to filter for genes of interest to display the InEx, the final product of the analysis. In case determining precise RBP binding sites is desired, we recommend performing CLIP-seq.^15^ For TTP, PAR-CLIP data has shown that both exonic (3′UTR) and intronic binding sites have a similar consensus sequence.^16^^,^^17^

The key conclusion that can be drawn from comparing the InEx value of enriched genes in PD versus Input is the preference of an RBP for binding to pre-mRNA or mature mRNA (Figure 2). This information reveals insights as to the phase of the RNA life cycle in which the specific RBP binds, a mechanistically important yet so far largely neglected aspect of RBP-RNA interactions.

## Limitations

Theoretically, the InEx values should be between 0 and 1. However, the calculated InEx occasionally has values above 1, due to technical imperfections. Some of these imperfections are related to library preparation and sequencing^18^ resulting in unequal RNA-seq coverage among the exons of a given gene. The part of a gene with much lower coverage due to these technical limitations will impact the TPM value which is averaged over any non-redundant exonic or intronic regions of the gene.

**Figure 3:**
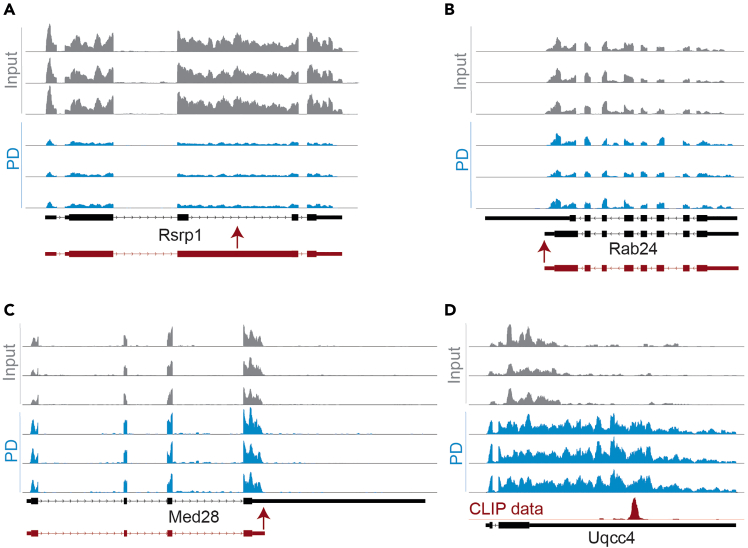
Annotation inaccuracy affects the InEx value RNA-seq fragment coverage tracks for three replicates of Input and PD are shown with annotations from the original reference genome shown in black (before adaption described in this protocol). Boxes in annotations are exons and connecting lines are introns, arrows on lines indicate the directionality of the gene. Red arrows indicate incongruencies between annotations and RNA-seq coverage and corrected putative annotations based on RNA-seq coverage are shown in red below the gene name. (A) Rsrp1 is shown as an example for retained introns that are not annotated as such. Coverage is in general much lower in introns than in exons. Intron 3, however, has equal coverage to exons, indicating it is retained in these cells. This falsely increases the quantity of intronic-covering fragments. (B and C) Rab24 and Med28 are shown as examples of genes with annotations where an exon extends further than the RNA-seq coverage, indicating that a more upstream polyadenylation signal is used in these cells. This can occur either in combination with (Rab24, in B) or without (Med28, in C) a more congruent variant annotated. Due to the averaging of coverage over exons and introns, a region annotated as exon without coverage decreases the exon quantification, thereby increasing the InEx. The discrepancies between annotations and RNA-seq coverage mentioned in A–C affect the InEx of either Input or PD samples in the same manner and are therefore negligible when comparing the InEx between Input and PD. (D) Uqcc4 is another example of an exon annotation extending further than RNA-seq coverage in Input. However, the pull-down enriched a longer transcript variant that covers the entire annotated exon. As in the depicted example, this can happen if the RBP binding site is only present in the longer variant. CLIP data used to infer the TTP binding site in this gene is from Sedlyarov et al.^16^ For this gene specifically, the InEx of Input and PD is therefore subject to different biases. Inferring a higher binding preference of TTP for pre-mRNA than mature mRNA from such InEx differences would therefore be invalid.

Another potential source of imperfections is incongruencies between the annotations in the reference genome and the expressed transcript variants (Figures 3A–3C). Retained introns that are not annotated as such can falsely increase the intronic TPM value (Figure 3A). Furthermore, multiple transcription start and/or termination sites in a gene can distort the estimation of the exon lengths needed for normalization (Figures 3B and 3C). Since these transcripts include only an extension of the first or last exon and thereby have at least one fragment covering each exon, these annotations are not excluded with the transcript filtering criteria applied here.

Lastly, the exact intron and exon length determination also depends on the positional precision (i.e. precise position of the exon/intron boundary) of the reference genome annotations.

While the latter sources of inaccuracies could be reduced by labor- and computationally intensive optimization, some inaccuracies for example due to sequencing efficiency, will likely remain. Therefore, the InEx values cannot serve as an absolute quantification of the percentage of pre-mRNA molecules in total mRNA molecules in a sample. However, when comparing the InEx values between conditions, all the above limitations will affect samples of either condition equally, essentially rendering them negligible. This holds true as long as the pull-down does not select for specific transcript variants, which could happen if the RBP binding site is only present in this variant (Figure 3D). In such cases, differentiating between transcript variants in the quantification of mature mRNA could be necessary to draw correct conclusions.

Lastly, while we assume that this protocol can be used with other RBPs, with some amendments even lncRNA binding proteins, we have not tested it for any other proteins besides TTP.

## Troubleshooting

### Problem 1

InEx inaccuracies due to discrepancies between annotations and fragment coverage (related to step 2).

### Potential solution

If further optimization is desired, the transcript filtering in step 2 could be substituted with transcript reconstruction algorithms. In that case, make sure to check the validity of predicted novel transcripts, especially when working with high sequencing depth.^19^ The issue of false prediction of novel transcripts has been addressed with a program that incorporates multiple transcript prediction algorithms, thereby achieving more accurate results than individual ones.^20^ Using this algorithm combined with steps 3–6 could provide an optimized unambiguous reference genome for intron and exon sequences.

### Problem 2

Fragment counts do not follow expected tendencies (related to step 7d).

### Potential solution

Check if the strandedness argument of the Htseq count command is in line with that of your library. This depends on the kit used for preparation, usually this information can be found in the kit’s specifications. In case you cannot find this information, you can benefit from having made stranded coverage tracks for checking your intermediate results throughout this protocol. If you set the same parameter of strandedness when creating your coverage tracks, you can check if your fragments are displayed on the tracks for the same strand the gene is annotated to be located on. If this is swapped or if you learn from looking up your kit that it was set incorrectly, correct the -s argument of Htseq count and, after running the corrected counting script again, check if the counts now follow expected trends.

### Problem 3

Many lowly expressed genes are found to be significantly enriched, especially in intronic counts, or filtering for genes with PD counts higher than average intronic PD counts removed too many genes (related to steps 11–13).

### Potential solution

The cutoff set here is only one of many options, so if you have too few or too many genes remaining, try adjusting it, keeping the following considerations in mind: (i) The log-transformation (step 12) spreads apart data for genes with low counts, making these genes more likely to be false positive due to random noise. Since mRNA is much more abundant than pre-mRNA, intronic counts in general are comparatively low and therefore more prone to be false positive. (ii) Log-Fold-Change cutoffs are often used to filter for genes with biologically relevant differential expression. However, the Log-Fold-Change is also higher with a lower initial expression level and setting such a cutoff would therefore further enrich for lowly expressed genes. Therefore, we set an absolute cutoff for counts to remove the false positives. (iii) Genes in this analysis are of interest when they are bound to the RBP and are therefore highly abundant in PD samples. Therefore, the filtering is applied on counts in PD samples. (iv) We chose to remove any genes that were below the average intronic counts in PD samples and applied the same cutoff for exonic counts in PD samples. However, this cutoff, as any such cutoffs, is arbitrary and you could optimize the filtering in your data by adjusting it.

## Resource availability

### Lead contact

Further information and requests for resources and reagents should be directed to and will be fulfilled by the lead contact, Pavel Kovarik (pavel.kovarik@univie.ac.at).

### Technical contact

Technical questions on executing this protocol should be directed to and will be answered by the technical contact, Christina Zeiler (christina.zeiler@univie.ac.at).

### Materials availability

This study did not generate new unique reagents.

### Data and code availability

• Raw RNA-seq data from Bestehorn et al.^1^ were deposited at the National Center for Biotechnology Information (NCBI)’s Sequence Read Archive (SRA) database under the bioproject identifier PRJNA1031176. CLIP-seq data from Sedlyarov et al.^16^ were deposited on Gene Expression Omnibus (GEO) under the reference series number GSE63468.
• The code used in this study is available at https://github.com/ChristinaZeiler/InEx.

## Acknowledgments

We acknowledge VBCF NGS Facility for RNA sequencing advice. The study was supported by the Austrian Science Fund grants W1261, P33000-B, and P35852 to P.K. We thank Daniel Malzl and Ronit Chakraborty for advice on general RNA-seq analysis methods.

## Author contributions

C.Z., A.B., and P.K. conceptualized the study. C.Z. and P.K. wrote the manuscript. C.Z. performed bioinformatics analysis and coding. A.B. performed pull-down and RNA-seq experiments.

## Declaration of interests

The authors declare no competing interests.
